# *Limnohabitans australis* sp. nov., isolated from a freshwater pond, and emended description of the genus *Limnohabitans*

**DOI:** 10.1099/ijs.0.022384-0

**Published:** 2010-12

**Authors:** Martin W. Hahn, Vojtěch Kasalický, Jan Jezbera, Ulrike Brandt, Karel Šimek

**Affiliations:** 1 Institute for Limnology, Austrian Academy of Sciences, Mondseestrasse 9, A-5310 Mondsee, Austria; 2 Biology Centre of the Academy of Sciences ČR, v.v.i., Institute of Hydrobiology, Na Sádkách 7, 37005 České Budějovice, Czech Republic; 3 Faculty of Science, University of South Bohemia, Branišovská 31, 37005 České Budějovice, Czech Republic

## Abstract

A chemo-organotrophic, aerobic, non-motile strain, MWH-BRAZ-DAM2D^T^, isolated from a freshwater pond in Brazil, was characterized phenotypically, phylogenetically and chemotaxonomically. Phylogenetic analysis of 16S rRNA gene sequences indicated affiliation of the strain with the genus *Limnohabitans* (*Comamonadaceae*, *Betaproteobacteria*). 16S rRNA gene sequence similarities between the isolate and *Limnohabitans* *curvus* MWH-C5^T^, representing the type species of the genus, and the type strains of *Limnohabitans parvus* and *Limnohabitans planktonicus* were 98.2, 96.5 and 97.0 %, respectively. DNA–DNA reassociation analyses with DNA of the type strains of all three previously described *Limnohabitans* species revealed similarity values in the range 26.2–44.6 %. The predominant fatty acids of the isolate were C_16 : 1_*ω*7*c*/*ω*6*c*, C_16 : 0_, C_12 : 0_ and C_8 : 0_ 3-OH, the major quinone was ubiquinone Q-8 and the DNA G+C content was 55.8 mol%. The isolate could be discriminated from the type strains of the three *Limnohabitans* species by several phenotypic traits including differences in the utilization of several carbon sources. Based on the phylogeny of the isolate and its differences from the three most closely related species, the isolate represents a novel species for which the name *Limnohabitans australis* sp. nov. is proposed. The type strain is MWH-BRAZ-DAM2D^T^ (=DSM 21646^T^=CCUG 56719^T^).

The genus *Limnohabitans* (*Comamonadaceae*, *Betaproteobacteria*) was established by Hahn *et al.* (2010) and currently contains three described species (Kasalický *et al.*, 2010). The genus represents a phylogenetic cluster formed mainly by environmental sequences obtained by cultivation-independent methods from numerous freshwater systems (e.g. Zwart *et al.*, 2002; Crump & Hobbie, 2005; Percent *et al.*, 2008; Shaw *et al.*, 2008), which was previously designated the ‘*Rhodoferax* sp. BAL47 cluster’ (Zwart *et al.*, 2002). Investigations using fluorescence *in situ* hybridization probes specific for the R-BT065 subcluster of the ‘*Rhodoferax* sp. BAL47 cluster’ (Šimek *et al.*, 2001), which is represented by two of the *Limnohabitans* species (Kasalický *et al.*, 2010), revealed the following relevant characteristics: a free-living, planktonic lifestyle (Šimek *et al.*, 2001); a significant contribution to total cell numbers of freshwater bacterioplankton (up to 30 %; Šimek *et al.*, 2010); a broad habitat range including, for instance, acidic and alkaline systems (Šimek *et al.*, 2010); and the potential for rapid growth under *in situ* conditions (Šimek *et al.*, 2006).

The type strains of all three *Limnohabitans* species described so far were all isolated from the water columns of stagnant freshwater systems located in Central Europe. Here, a novel strain, isolated from a pond located in subtropical South America, which is closely related to previously described *Limnohabitans* species, as well as to numerous uncultured bacteria represented by environmental sequences, is characterized and it is proposed that this strain represents a novel species, *Limnohabitans australis* sp. nov.

Strain MWH-BRAZ-DAM2D^T^ was isolated from the subtropical Monjolinho Pond (2 ° 59′ 10.14″ S 4 ° 52′ 50.28″ W) located on the university campus in São Carlos, São Paulo state, Brazil. The strain was isolated by using the dilution-acclimatization method and NSY medium (Hahn *et al.*, 2004, 2005). Strain MWH-BRAZ-DAM2D^T^ grew on a variety of solidified complex media including Luria–Bertani agar (Difco BD), Casitone agar (Difco BD), R2A agar (Remel) and NSY agar (Hahn *et al.*, 2004), forming unpigmented, smooth, convex colonies.

Carbon source utilization tests and other phenotypic characterizations were performed as described previously (Hahn *et al.*, 2009). Briefly, growth enabled by utilization of specific substrates was determined by comparison of optical density established in liquid one-tenth-strength NSY medium (0.3 g l^−1^) with and without the respective test substrate (0.5 g l^−1^). Optical density differences of <10 %, 10–50 % and >50 % of the optical density established on medium without test substrate were scored after 10 days of growth as no utilization (−), weak utilization (w) and good utilization (+), respectively. Analysis of the phylogenetic position of the novel isolate was performed by 16S rRNA gene sequence analysis as described previously (Hahn *et al.*, 2009). Determination of the DNA G+C content, analysis of major respiratory lipoquinones and DNA–DNA reassociation experiments, to ascertain whether the novel isolate belongs to a previously described *Limnohabitans* species (Wayne *et al.*, 1987), were all carried out by the Identification Service and B. J. Tindall, DSMZ, Braunschweig, Germany. Fatty acid profiles were characterized by using the MIS Sherlock automatic identification system (MIDI) and the Sherlock Aerobic Bacterial Database (TSBA60) as described by Greenblatt *et al.* (1999). Biomass of duplicate cultures obtained by growing the strain in NSY medium (3 g l^−1^) for 2 days at 21 °C was analysed. Results of the phenotypic and chemotaxonomic investigations are presented in Tables 1 and 2.

blast searches (Altschul *et al.*, 1990) against the database with the 16S rRNA gene sequence of the novel isolate in December 2009 resulted in five and 81 hits with >99 % and >97 % sequence similarities, respectively. Thirteen out of the 81 hits represented cultivated strains, whereas the majority represented uncultivated bacteria. No cultivated strains were among the hits with >99 % sequence similarity. This group was represented by environmental sequences obtained from two estuary systems (Delaware and Chesapeake Bays, USA), as well as from Ipswich River, MA, USA (Shaw *et al.*, 2008; Crump & Hobbie, 2005). A phylogenetic analysis of the relationship of cultivated *Limnohabitans* strain and environmental sequences representing the so-called ‘*Rhodoferax* sp. BAL47 cluster’ was presented previously (Hahn *et al.*, 2010).

Phylogenetic analyses with sequence sets representing the most closely related recognized species by using the neighbour-joining (NJ) and the maximum-likelihood (ML) methods consistently revealed the affiliation of strain MWH-BRAZ-DAM2D^T^ with the genus *Limnohabitans* (Fig. 1). The 16S rRNA gene sequence of the isolate possessed sequence similarities of 98.2, 97.0 and 96.5 % to those of the type strains of *Limnohabitans curvus*, *Limnohabitans parvus* and *Limnohabitans* *planktonicus*, respectively. DNA–DNA reassociation analyses with DNA of the type strains of the three previously described *Limnohabitans* species resulted in similarity values (mean values of duplicate measurements) of 26.2 % (*L. parvus*), 30.0 % (*L. planktonicus*) and 44.6 % (*L. curvus*). The duplicate measurements performed for each of the three pairings differed by 2.6 % (*L. curvus*) to 5.0 % (*L. planktonicus*). The predominant fatty acids of the isolate were C_16 : 1_*ω*7*c*/*ω*6*c* (73.5 %), C_8 : 0_ 3-OH (8.2 %), C_16 : 0_ (7.7 %) and C_12 : 0_ (7.4 %). The major quinone was ubiquinone Q-8 and the DNA G+C content was 55.8 mol% (Tables 1 and 2).

Strain MWH-BRAZ-DAM2D^T^ can be distinguished from the type strain of *L. curvus* (Hahn *et al.*, 2010) by its ability to utilize malonate and its inability to utilize ethanol, propionate, malate, citrate, d-ribose, d-galactose, d-mannose and sucrose, as well as by a lower maximum NaCl concentration that supports growth and differences in the minimum and maximum growth temperatures (Table 1). In addition, the two strains differ in the presence of minor fatty acid compounds (Table 2). Differential traits that separate strain MWH-BRAZ-DAM2D^T^ from all three previously described *Limnohabitans* species (Hahn *et al.*, 2010; Kasalický *et al.*, 2010) are utilization of malonate and no utilization of malate or citrate, as well as the absence of the minor fatty acids C_16 : 1_*ω*5*c*, C_18 : 0_, 11-Me-C_18 : 1_*ω*7*c* and C_18 : 1_*ω*9*c*, a catalase-negative reaction, a lower maximum NaCl concentration that supports growth and higher minimum and maximum growth temperatures (Tables 1 and 2). Differences in thermal adaptation may reflect differences in adaptation to local climate conditions at the sites of origin of the four strains (subtropical versus temperate climate) and may not represent a trait shared by all members of the proposed species *L. australis* sp. nov. (Hahn & Pöckl, 2005).

The phylogenetic analysis, as well as several phenotypic and chemotaxonomic similarities, suggest that strain MWH-BRAZ-DAM2D^T^ belongs to the genus *Limnohabitans*. The results of the DNA–DNA reassociation analyses demonstrate that the strain does not belong to one of the previously described *Limnohabitans* species when the recommendation of a threshold value of 70 % DNA–DNA similarity for delineation of prokaryotic species (Wayne *et al.*, 1987) is considered. Therefore, it is proposed that the novel species *Limnohabitans australis* sp. nov. be established to accommodate strain MWH-BRAZ-DAM2D^T^.

## Emended description of the genus *Limnohabitans* Hahn *et al.* 2010 emend. Kasalický *et al.* 2010

The description of the genus *Limnohabitans* is as given previously (Hahn *et al.*, 2010; Kasalický *et al.*, 2010), but with the following amendment. Members of the genus can be catalase-positive or catalase-negative.

## Description of *Limnohabitans australis* sp. nov.

*Limnohabitans australis* (aus.tra′lis. L. masc. adj. *australis* southern, relating to the region in which the organism was isolated).

Cells are curved rods, 1.0–1.7 μm in length and 0.4–0.5 μm in width. Chemo-organotrophic, aerobic, facultatively anaerobic, oxidase-positive and catalase-negative. Colonies grown on NSY agar are unpigmented, circular and convex with a smooth surface. Growth occurs at 12–36 °C and with 0–0.2 % (w/v) NaCl. Assimilates acetate, glycerate, *α*-ketoglutarate, pyruvate, succinate and gluconate. Weak assimilation of malonate, fumarate, glyoxylate and glucose. No assimilation of several substrates (Table 1). Major cellular fatty acids (>5 % of total) are C_12 : 0_, C_16 : 0_, C_16 : 1_*ω*7*c*/*ω*6*c* and C_8 : 0_ 3-OH. The major quinone is ubiquinone Q-8.

The type strain is MWH-BRAZ-DAM2D^T^ (=DSM 21646^T^=CCUG 56719^T^), isolated from Monjolinho Pond, São Carlos, Brazil. The DNA G+C value of the type strain is 55.8 mol%.

## Figures and Tables

**Fig. 1.**
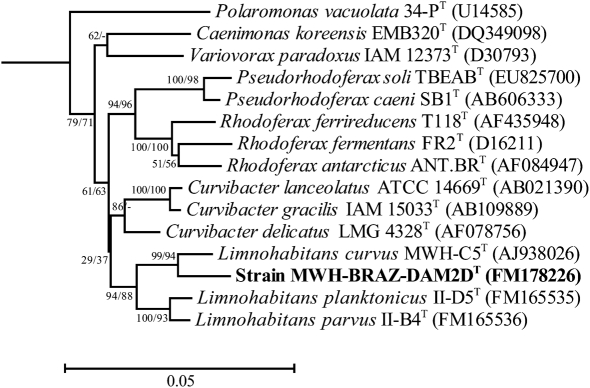
NJ tree (1000 bootstrap iterations) based on almost complete 16S rRNA gene sequences showing the phylogenetic position of strain MWH-BRAZ-DAM2D^T^. An ML tree (100 bootstrap iterations; not shown) calculated with the same sequence set revealed almost identical branching orders to those in the NJ tree. Bootstrap values obtained by the NJ (first value) and ML (second value) methods are presented. Nodes not reconstructed in the ML tree show a horizontal slash instead of a bootstrap value. Bar, 5 nt substitutions per 100 nt.

**Table 1.** Phenotypic traits of strain MWH-BRAZ-DAM2D^T^ (*Limnohabitans australis* sp. nov.) and other members of the genus *Limnohabitans* Strains: 1, *Limnohabitans australis* sp. nov. strain MWH-BRAZ-DAM2D^T^; 2, *L. curvus* strain MWH-C5^T^ (Hahn *et al.*, 2010); 3, *L. parvus* strain II-B4^T^ (Kasalický *et al.*, 2010); 4, *L. planktonicus* strain II-D5^T^ (Kasalický *et al.*, 2010). Substrate utilization tests were performed for all four strains under the same conditions. All four strains grew under anoxic conditions, were oxidase-positive, non-motile and unpigmented, and possessed ubiquinone Q-8 as major quinone. Furthermore, all strains were positive for utilization of d-glycerate, butyrate and *α*-ketoglutarate and none of the four strains utilized oxalate, dl-lactate, l-arginine, l-sorbose, *N*-acetylglucosamine, l-carnitine, betaine or spermidine. −, Negative; +, positive; w, weakly positive.

**Characteristic**	**1**	**2**	**3**	**4**
Cell morphology	Curved rods	Curved rods	Short rods	Rods
Cell length (μm)	1.0–1.7	1.0–1.5	0.6	0.9
Cell width (μm)	0.4–0.5	0.4–0.5	0.3	0.3
Growth temperature				
Minimum (°C)	12 (w)	4	4 (w)	4
Maximum (°C)	36	34	34	34
Maximum NaCl concentration (%)	0.2	0.5	0.5	0.5
Catalase	**−**	**+**	**+**	**+**
Utilization of:				
Ethanol	**−**	w	**−**	w
Glycerol	**−**	**−**	w	**+**
Glyoxylate	w	**−**	**−**	w
Glycolate	**−**	**−**	**−**	w
Acetate	**+**	**+**	**−**	**+**
Propionate	**−**	w	**−**	**−**
Pyruvate	**+**	**+**	w	**+**
dl-Malate	**−**	**+**	**+**	**+**
Malonate	w	**−**	**−**	**−**
Oxaloacetate	**−**	**−**	**−**	**+**
Succinate	**+**	**+**	w	**+**
Fumarate	w	**+**	w	**+**
Citrate	**−**	**+**	**+**	**+**
l-Glutamate	**−**	**−**	**+**	**+**
l-Glutamine	**−**	**−**	w	**+**
l-Histidine	**−**	**−**	**−**	**+**
l-Phenylalanine	**−**	**−**	**−**	**+**
l-Proline	**−**	**−**	**+**	**+**
l-Serine	**−**	**−**	**−**	**+**
l-Tryptophan	**−**	**−**	**+**	w
d-Ribose	**−**	w	**−**	**−**
d-Glucose	w	**+**	**+**	**+**
d-Galactose	**−**	w	**−**	**−**
d-Mannose	**−**	**+**	w	**+**
Sucrose	**−**	w	**−**	**−**
d-Gluconate	**+**	**+**	**−**	**−**
DNA G+C content (mol%)	55.8	55.5	59.9	59.9

**Table 2.** Whole-cell fatty acid composition of *Limnohabitans australis* sp. nov. and other members of the genus *Limnohabitans* Strains: 1, *Limnohabitans australis* sp. nov. strain MWH-BRAZ-DAM2D^T^; 2, *L. curvus* strain MWH-C5^T^ (Hahn *et al.*, 2010); 3, *L. parvus* strain II-B4^T^ (Kasalický *et al.*, 2010); 4, *L. planktonicus* strain II-D5^T^ (Kasalický *et al.*, 2010). All strains were cultivated under identical conditions [NSY medium (3 g l^−1^) at 21 °C for 2 days]. The presented data are percentages of the summed fatty acids and represent mean values from analysed duplicate cultures. nd, Not detected.

**Fatty acid**	**1**	**2**	**3**	**4**
C_8 : 0_ 3-OH	8.2	2.7	1.0	0.7
C_10 : 0_ 3-OH	nd	nd	nd	1.5
C_12 : 0_	7.4	4.5	3.6	2.9
C_12 : 0_ 3-OH	nd	nd	1.8	nd
C_14 : 0_	1.0	1.0	0.4	0.5
C_14 : 1_*ω*5*c*	0.6	0.4	0.2	0.2
C_15 : 1_*ω*6*c*	nd	nd	1.3	nd
C_16 : 0_	7.7	14.0	15.0	19.5
C_16 : 1_*ω*5*c*	nd	0.2	0.5	0.7
C_16 : 1_*ω*7*c*/C_16 : 1_*ω*6*c*	73.5	76.7	66.4	62.4
C_17 : 0_	nd	nd	1.3	nd
C_17 : 0_ cyclo	nd	nd	nd	0.7
C_17 : 1_*ω*6*c*	nd	nd	2.6	nd
C_18 : 0_	nd	0.3	0.5	0.3
11-Me-C_18 : 1_*ω*7*c*	nd	0.3	nd	1.3
C_18 : 1_*ω*7*c*/C_18 : 1_*ω*6*c*	1.7	1.8	5.3	8.9
C_18 : 1_*ω*9*c*	nd	0.2	0.3	0.5
